# Association between Stereotactic Radiotherapy and Death from Brain Metastases of Epithelial Ovarian Cancer: a Gliwice Data Re-Analysis with Penalization

**DOI:** 10.22034/APJCP.2017.18.4.1113

**Published:** 2017

**Authors:** Andrzej Tukiendorf, Mohammad Ali Mansournia, Jerzy Wydmański, Edyta Wolny-Rokicka

**Affiliations:** 1 *Department of Epidemiology and Silesia Cancer Registry,*; 3 *Department of Radiotherapy, Cancer Center-Institute of Oncology, ul. AK 15, 44-101 Gliwice,*; 4 *Department of Radiotherapy, Regional Clinical Hospital, ul.Zyty 26, 65-001 ZielonaGóra, Poland, *; 2 *Department of Epidemiology and Biostatistics, School of Public Health, Tehran University of Medical Sciences, Tehran, Iran. *

**Keywords:** Small number datasets, sparse data bias, penalized Cox regression

## Abstract

**Background:**

Clinical datasets for epithelial ovarian cancer brain metastatic patients are usually small in size. When adequate case numbers are lacking, resulting estimates of regression coefficients may demonstrate bias. One of the direct approaches to reduce such sparse-data bias is based on penalized estimation.

**Methods:**

A re- analysis of formerly reported hazard ratios in diagnosed patients was performed using penalized Cox regression with a popular SAS package providing additional software codes for a statistical computational procedure.

**Results:**

It was found that the penalized approach can readily diminish sparse data artefacts and radically reduce the magnitude of estimated regression coefficients.

**Conclusions:**

It was confirmed that classical statistical approaches may exaggerate regression estimates or distort study interpretations and conclusions. The results support the thesis that penalization via weak informative priors and data augmentation are the safest approaches to shrink sparse data artefacts frequently occurring in epidemiological research.

## Introduction

Brain metastases (BMs) as a late manifestation of epithelial ovarian cancer (EOC) are rare (following different reports from one to a few percent of cases (Cohen et al, 2005; Pectasides et al, 2005; Pietzner et al, 2009), but the diagnosis of its occurrence has been increasing in recent years (Hardy and Harvery, 1989; Bruzzone et al, 1993; Chiang et al, 2012) probably owing to more effective treatment of the primary cancer and the resulting prolongation of survival (Cohen et al, 2005).

In the last few years stereotactic radiotherapy (SRT) has come into focus as a promising therapy option in brain metastases from ovarian cancer and it has been shown that prompt stereotactic radiosurgery is advantageous in EOC BM patients (Pietzner et al, 2009). Some studies (Lee et al, 2007) describe the observed remarkable median survival after treatment with SRT in contrast with whole brain radiotherapy (WBRT). Supported by the previous studies, SRT has become increasingly popular (Gadducci et al, 2007) as another promising therapy option or even optimal treatment (Brown et al, 2005; Navarro-Martín et al, 2009). Since EOC BM is a rare clinical event, the analyzed datasets comprised usually a small number of patients, for example, 23 patients in the Milano study (Cormio et al, 1995), 10 patients in the Taipei case (Chen et al, 2011), or 32 patients in the Gliwice database (Celejewska et al, 2014).

Ratio measures (such as risk ratios, odds ratios, and hazard ratios) that are commonly used to quantify the effect of a treatment or other factors on an event outcome using maximum likelihood methods, assume that the number of events observed is sufficient to result in well-adjusted estimates. Unfortunately, when the data lack adequate case numbers, the resulting estimates of the regression coefficients can have a bias (often known as sparse data bias). This bias is sometimes called a ‘small sample bias’ but in fact it can occur in quite large datasets. Thus, it is better termed as sparse data bias (Sullivan and Greenland, 2013; Discacciati et al, 2015; Greenland and Mansournia, 2015; Greenland et al, 2016).

One of the direct approaches to reduce the sparse-data bias is based on the penalized estimation (i.e. a form of shrinkage estimation), in which weakly informative priors can easily diminish sparse data artefacts without requiring excessive contextual information. What is more, penalization can be easily performed with common packages like SAS, Stata and R (Sullivan and Greenland, 2013; Discacciati et al, 2015; Greenland and Mansournia, 2015; Greenland et al, 2016). These studies also provide extra software codes for computational statistical procedures based on the described examples. 

Confidently, in the authors’ previous study on the same topic (Celejewska et al, 2014) the sparse data bias could be noted as the effect of the interval to SRT on the survival in patients, and the published results slightly exceeded the line with sensible expectations. Application of the Weibull regression in a classical and Bayesian approaches resulted in the estimated HRs (95% CI) at the level of 20 (6, 67), and 28 (5, 89) – see (Celejewska et al, 2014). This indicated that the risk of death in late SRT patients is 20 or 28 times greater than in earlier treated population. Following penalized methods (Sullivan and Greenland, 2013; Discacciati et al, 2015; Greenland and Mansournia, 2015; Greenland et al, 2016), preferably called Boxian statistics (Greenland, 2016) we made efforts to spot a less dramatic bias.

The aim of this study is to estimate the hazard ratio between SRT and death from brain metastases of epithelial ovarian cancer using penalized Cox regression.

## Material and Methods

The analyzed dataset included 32 patients who were diagnosed with BM from EOC and underwent SRT in the Cancer Center and Institute of Oncology in Gliwice, Poland, between 2003 and 2013 (with a prior EOC diagnosis conducted since 1998). More detailed characteristics of patients have been described in (Celejewska et al, 2014). Table 1 presents the full dataset.

**Figure 1:**
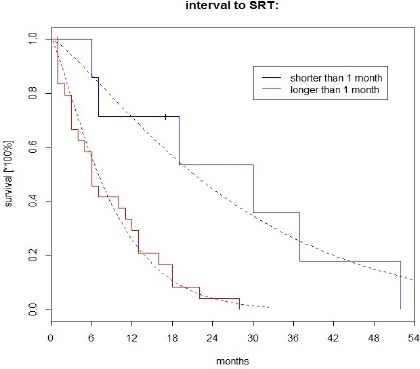
Survival Since BM Diagnosis (Cox Regression)

**Table 1:** Analyzed Dataset (BMs Survival in EOC Patients)

patient	BMFS	no. of BMs	WBRT	SRT	survival	censored
1	22	1	0	0	7	1
2	51	2	0	0	19	1
3	30	2	1	1	12	1
4	25	2	0	1	1	1
5	24	2	0	0	30	1
6	27	1	1	1	13	1
7	31	1	1	1	22	1
8	118	3	0	1	1	1
9	67	1	0	1	7	1
10	93	3	1	1	3	1
11	11	1	1	1	18	1
12	30	2	1	1	13	1
13	37	1	0	1	3	1
14	15	2	0	1	2	1
15	97	2	0	1	3	1
16	6	3	1	1	10	1
17	153	2	1	1	1	1
18	31	1	1	1	6	1
19	22	3	1	1	18	1
20	67	1	1	1	1	1
21	12	1	0	0	37	1
22	15	3	1	1	4	1
23	44	2	0	0	6	1
24	22	2	0	1	6	1
25	29	3	1	1	6	1
26	28	2	1	1	5	1
27	15	2	1	1	16	1
28	49	1	0	0	52	1
29	27	1	1	1	11	1
30	34	1	1	1	28	1
31	68	1	0	0	1	0
32	24	1	0	0	17	0

**Table 2:** Hazard Ratios of the SRT Risk Factor (Univariate Analysis)

method	HR	95% CI	p-value
Cox regression	5.57	(1.63, 19.09)	0.0062
discrete-time hazard model	3.51	(1.40, 8.79)	0.0077

**Table 3:** Hazard Ratios (Multivariate Analysis)

Method	risk factor	HR	(95% CI)	p-value
Cox regression	BMFS	1.02	(1.01, 1.04)	0.0337
	no. of BMs	1.85	(1.10, 3.12)	0.0204
	WBRT	0.28	(0.09, 0.90)	0.0322
	SRT	17.6	(3.5, 88.6)	0.0005
discrete-time hazard model	BMFS	1.02	(1.01, 1.04)	0.0286
	no. of BMs	1.97	(1.12, 3.49)	0.0197
	WBRT	0.21	(0.06, 0.73)	0.0133
	SRT	26.3	(4.70, 149)	0.0002
penalized Cox regression	BMFS	1.02	(1.01, 1.03)	0.0466
	no. of BMs	1.72	(1.02, 2.89)	0.0384
	WBRT	0.36	(0.13, 0.96)	0.0394
	SRT	10.9	(4.47, 27.1)	<0.0001

**Figure 2:**
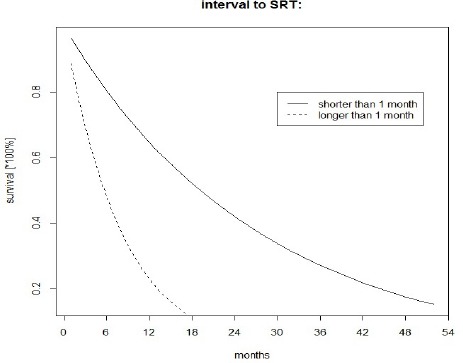
Survival Since BM Diagnosis (Discrete-Time Hazard Model)

In Table 1, the ‘BMFS’ abbreviation stands for the brain metastases free survival measured in months since EOC diagnosis, while ‘SRT’ is the interval to stereotactic radiotherapy (longer than 1 month: 1, shorter than 1 month: 0). The survival is the time to death of patients since BM diagnosis also measured in months (censored stands for occurrence of the event). 

Relationships between possible risk factors and survival after SRT were assessed using statistical methodology. Classical Cox regression and discrete-time hazard model (Singer and Willett, 2003) were applied in the statistical analysis. First, to detect possible sparse data artefact, a univariate regression was conducted for the ‘SRT’ risk factor and its survival curves were presented graphically. Then, to shrink the ‘SRT’ risk factor coefficient estimate, a multivariable penalized Cox regression was additionally performed using data augmentation prior. A prior CI 95% interval of 1/8 to 8 were assumed for ‘SRT’ (for details see Sullivan and Greenland, 2013). The SAS software codes are printed in the Appendix. 

## Results

The computed HRs of the SRT risk factor in univariate Cox regression and discrete-time hazard model are reported in Table 2.

The survival curves for the time since BM diagnosis following the interval to SRT are presented in Figures 1 and 2. 

From the results presented above (Table 2, Figures 1 and 2), any ‘exaggerated’ statistical impact of the interval to SRT on the survival in patients can be found in the estimated regression coefficients and survival curves. In turn, the estimated HRs in the multivariate analysis are given in Table 3.

Following the estimates in Table 3, an apparent reduction of HR for the ‘SRT’ was established using penalized Cox regression, roughly by a half in comparison with a classical Cox regression and discrete-time hazard model. Since no priors were assumed for ‘BMFS’, ‘no. of BMs’, and ‘WBRT variables, the estimates of the parameters did not change statistically in these models and as a consequence, they look similar (see Table 3 HR estimates).

## Discussion

Considering the above mentioned and HRs reported in Table 2, no probable evidence of the sparsity in data can be assessed, nor can be the ‘disturbing’ effect of the ‘SRT’ on the clinical event. The survival curves shown in Figures 1 and 2 also look ‘typical’ and nothing ‘unusual’ can be concluded from these simple models. However, the situation changes radically in a multivariate approach. A strong increase of the influence of the ‘SRT’ on death of patients is noticed (similarly as in Celejewska et al, 2014). This is rather a statistical consequence than the clinical exact cause-effect relationship (a clear explanation can be found in (Sullivan and Greenland, 2013; Discacciati et al, 2015; Greenland and Mansournia, 2015; Greenland et al, 2016). 

The adopted statistical methodology of the penalized regression radically changed the strength of a plausible impact of the analyzed risk factor on the estimated survival. Even though this conclusion stands for self-criticism of the previously reported results (Celejewska et al, 2014), it is worth noting for certain reasons. From a medical and clinical point of view, absently adopted clinical reports by medical doctors and practitioners without properly conducted methodological evaluation may be even detrimental to patients’ cure. A similar remark about the ignorance of time-dependent confounders for the effect of physical activity on functional performance and knee pain in patients with osteoarthritis can be found in (Mansournia et al, 2012). 

Moreover, the final outcome confirms that even weak informative priors can substantially diminish sparse data bias, which is prevailing in medical research, following our research experience. Hence, the so-called Boxian statistics proposed by Greenland (Greenland S, 2016) with a wide range of penalized methods seem to be a rational computational alternative providing better interpretation of treatment results and health benefits.

The paper elaborates on the discrepancies between the present and the formerly obtained results and provides an extension to the statistical background of the epidemiological methodology for sparse data bias. Based on the above, the following conclusions can be drawn: 

• Sparse data problem often appears in medical data, especially in small sample sets 

• Classical statistical approaches may exaggerate the regression estimates and distort the study conclusions 

• Using penalization via data augmentation is the easiest and safest approach to diagnose and solve the sparse data artefacts.

• Penalized statistics can easily diminish a plausible impact of expectation to stereotactic radiosurgery on the survival in patients with epithelial ovarian cancer brain metastases and provide a rational alternative to improve interpretation of data with a sparse bias 

• A wider clinical discussion is required on the problem discovered.
